# Retraction Note: MicroRNA-454 regulates stromal cell derived factor-1 in the control of the growth of pancreatic ductal adenocarcinoma

**DOI:** 10.1038/s41598-023-48852-5

**Published:** 2023-12-06

**Authors:** Yue Fan, Li-Li Xu, Chen-Ye Shi, Wei Wei, Dan-Song Wang, Ding-Fang Cai

**Affiliations:** 1 Department of Integrated TCM & Western Medicine, Zhongshan Hospital, Fudan University, Shanghai, 200032 China; 2 Department of General Surgery, Zhongshan Hospital, Fudan University, Shanghai, 200032 China; 3 Shanghai Institutes for Biological Sciences, Chinese Academy of Sciences, Institute for Nutritional Sciences, Shanghai, 200032 China

Retraction of: *Scientific Reports* 10.1038/srep22793, published online 15 March 2016

The Editors have retracted this Article.

After publication, concerns were raised that three tumour images in Figure 4E appear to be identical:

- PANC-1-null far-left image;
- PANC-1-miR-454 middle image;
- PANC-1-miR-454 far-right image.

In addition, panel CD68/NT in Figure 1A overlaps with panel CD45/PDL-Day 0 of Figure 3A in^1^.

Ding-Fang Cai has not responded to any correspondence from the Editors about this retraction. The Editors were unable to obtain current email addresses for Yue Fan, Li-Li Xu, Chen-Ye Shi and Dan-Song Wang. Wei Wei has stated that they were not aware of the submission of this article.
